# Impact of an interdisciplinary standard operating procedure on external ventricular drainage-associated ventriculitis and antibiotic use in intensive care unit patients: a retrospective pre–post study

**DOI:** 10.1186/s44158-026-00409-4

**Published:** 2026-05-23

**Authors:** Michaela Danassis, Nilufar Akbari, Hans-Jörg Epple, Inna Friesen, Nils Hecht, Sonja Hansen, Sascha Treskatsch, Stefan Angermair

**Affiliations:** 1 Department of Anaesthesiology and Intensive Care Medicine, Charité–University Medicine Berlin, Corporate Member of Freie Universität Berlin and Humboldt-Universität zu Berlin, Campus Benjamin Franklin, Berlin, Germany; 2 Institute of Biometry and Clinical Epidemiology, Charité–University Medicine Berlin, Corporate Member of Freie Universität Berlin and Humboldt-Universität zu Berlin, Berlin, Germany; 3 Antibiotic Stewardship, Charité–University Medicine Berlin, Corporate Member of Freie Universität Berlin and Humboldt-Universität zu Berlin, Berlin, Germany; 4 National and Supranational Reference Center for Mycobacteria, Research Center Borstel, Borstel, Germany; 5 Department of Neurosurgery, Charité–University Medicine Berlin, Corporate Member of Freie Universität Berlin and Humboldt-Universität zu Berlin, Berlin, Germany; 6 Institute for Hygiene and Environmental Medicine, Charité–University Medicine Berlin, Corporate Member of Freie Universität Berlin and Humboldt-Universität zu Berlin, Berlin, Germany

**Keywords:** Ventriculitis, External ventricular drainage, Antibiotic stewardship, Intensive care unit, Neurocritical care

## Abstract

**Background:**

External ventricular drainage (EVD)-associated ventriculitis is a serious complication in neurocritical care. Diagnostic uncertainty and heterogeneous infection prevention and control (IPC) practices may contribute to variable ventriculitis rates and potentially avoidable antibiotic exposure. We evaluated the impact of implementing an interdisciplinary standard operating procedure (SOP) on ventriculitis incidence and antimicrobial stewardship (ABS)-related outcomes.

**Methods:**

We conducted a retrospective single-centre pre–post study in one intensive care unit (ICU) at Charité–Universitätsmedizin Berlin (2019–2023). Adult patients with EVDs were assigned to a pre-SOP cohort (Group 1) or a post-SOP cohort (Group 2). The SOP comprised standardized IPC protocols, an algorithm-based diagnostic workup and evidence-based anti-infective strategies. The primary endpoint was study-defined EVD-associated ventriculitis, adjudicated retrospectively using uniform criteria. Secondary endpoints included empirical antibiotic initiation, duration of therapy, antibiotic consumption, ICU length of stay (LOS), mortality, and SOP adherence. Cerebrospinal fluid (CSF) parameters and clinical signs were summarized descriptively.

**Results:**

A total of 166 patients were included (pre-SOP, Group 1: *n* = 50; post-SOP, Group 2: *n* = 116). The study-defined ventriculitis rate decreased from 22% [95% CI 0.13–0.35] to 9.5% [95% CI 0.05–0.16] with an unadjusted OR 0.37 [95% CI 0.13–1.04]; *p* = 0.053, attenuated with an adjusted OR of 0.50 [95% CI 0.19–1.35]; *p *= 0.169.

Incidence density declined from 21.1 to 12.0 per 1000 EVD days. Empirical antibiotic use fell from 28.0% [95% CI 17.5–41.7] to 12.9% [95% CI 8.0–20.2], corresponding to an unadjusted OR of 0.38 [95% CI 0.17–0.87] and median therapy duration decreased from 16 [95% CI 13.5–18.5] to 10 days [95% CI 6.5–13.5]. ICU LOS shortened by 4 days, while ICU mortality remained unchanged (26% vs. 27%).

**Conclusion:**

An interdisciplinary SOP was associated with reduced empirical antibiotic exposure and shorter treatment duration without affecting ICU mortality. The SOP appeared to improve diagnostic consistency and standardization of the workup rather than diagnostic test performance.

**Trial registration:**

The study is registered in the German Clinical Trial Register (DRKS ID: 00036075) in February 2025.

**Supplementary Information:**

The online version contains supplementary material available at 10.1186/s44158-026-00409-4.

## Introduction

External ventricular drainage (EVD) is essential in neurosurgical and intensive care management of conditions such as subarachnoid haemorrhage, traumatic brain injury and hydrocephalus [1–3]. However, EVDs carry a considerable risk of infection with ventriculitis rates reported between 2 and 24% [4–6]. Risk factors include intraventricular haemorrhage, prolonged drainage (> 5–7 days), inadequate aseptic handling and repeated system manipulations [7–10]. EVD-associated ventriculitis increases morbidity, prolongs intensive care unit (ICU) length of stay (LOS) and may worsen outcomes, including mortality [1, 7].

Differentiating true infection from sterile postoperative inflammation or contamination remains a major diagnostic challenge [9–12]. Clinical symptoms are often nonspecific and cerebrospinal fluid (CSF) analysis can be confounded by blood contamination or prior interventions [10, 11].

To address these challenges, a multidisciplinary team at Charité–Universitätsmedizin Berlin developed and implemented a standardized EVD-standard operating procedure (SOP). The SOP integrates infection prevention and control (IPC) strategies, diagnostic algorithms and antimicrobial stewardship (ABS) principles. We hypothesized that this approach would reduce nosocomial EVD-associated ventriculitis rates, improve diagnostic precision, lower empirical antibiotic use and shorten ICU stays—ultimately enhancing patient outcomes and minimizing unnecessary antimicrobial exposure.

## Materials and methods

### Data collection

Patient data for case identification were retrieved through Structured Query Language (SQL) inquiries using the hospital’s electronic medical record system (COPRA System GmbH, Sasbachwalden, Germany, and SAP AG, Walldorf, Germany).

Only data from patients’ index ICU stay were included; no post-ICU follow-up was conducted.

### Study design and patients

The study was conducted as a single-centre, non-randomized, retrospective pre-post analysis. Adult patients (≥ 18 years) at their index stay being treated with an EVD in a mixed 30-bed ICU at Charité–Universitätsmedizin Berlin, Campus Benjamin Franklin, between January 2019 and December 2023, were included. Patients were excluded if they experienced pre-existing meningitis on ICU admission or if the EVD remained in place for less than 3 days.

### Intervention

The SOP introduced a standardized diagnostic and therapeutic algorithm, predefined IPC measures and structured antimicrobial stewardship principles. A multidisciplinary collaboration involved several Departments at Charité. In collaboration with the Institute of Hygiene and Environmental Medicine, IPC protocols were defined for both the placement and ongoing management of EVDs. The ABS team designed the antibiotic prophylaxis and treatment regime. Pathogen identification and interpretation of antibiotic susceptibility testing was performed by the Institute of Microbiology at Labor Berlin. The Department of Neurosurgery standardized the technical procedure for EVD insertion and contributed to defining the frequency and interpretation of CSF analysis. The Department of Anaesthesiology and Intensive Care Medicine provided expertise in ICU-specific infection control and overall patient management.

The SOP was not formally validated but was developed through expert consensus using a structured internal Delphi process. Finalized in June 2021, it was introduced within a dedicated three-month implementation phase including staff training and supervision to IPC measures and a diagnostic and therapeutic algorithm. The SOP was made permanently accessible via the hospital’s internal information system.

The diagnostic and therapeutic algorithm is presented in the Supplementary Fig. 1.

Key differences between pre- and post-SOP practice are summarized in Table 1.

**Table 1:** Comparison between measures pre- and post-SOP implementation

Key aspects	Procedure pre-SOP	Procedure post-SOP
**1. Neurosurgical**		
• **Preoperative single-shot antibiotics prophylaxis**	**Varies depending on the provider**	**Standard with cefazolin**
• Aseptic technique during EVD • insertion in accordance to neurosurgical standards	Standard	Standard
• Proper EVD catheter tunnelling through the skin, sterile dressing of the insertion site	Standard	Standard
**2. IPC measures**		
• Type of EVD catheters	Non-antimicrobial-impregnated EVD catheters, closed catheter system with a dedicated access port	Non-antimicrobial-impregnated EVD catheters, closed catheter system with a dedicated access port
• **Hygienic hand disinfection**	**Varies depending on the provider**	**Hygienic hand disinfection before any manipulation of the catheter system (CSF sampling or therapeutic CSF drainage)**
• Skin antisepsis at EVD insertion and for dressing changes	Octenidine-based solution	Octenidine-based solution
• Dressing type	Non-antimicrobial	Non-antimicrobial
• **Wound inspection and dressing changes**	**Varies depending on the provider**	**Wound inspection and dressing changes once daily**
• **Aseptic CSF sampling**	**Varies depending on the provider**	**Strictly under aseptic conditions via the access port of the closed system, which was disinfected prior to sampling with the recommended contact time Standard after training skills**
**3. Diagnostic and monitoring**		
• **Indication for CSF sampling**	• **Baseline at EVD insertion time (CSF 1 st)** • **Triggered by clinical suspicion or fever (CSF 2nd)** • **Monitoring treatment response or further diagnostic clarification (CSF 3rd)**	• **Baseline at EVD insertion time (CSF 1 st)** • **Triggered by clinical suspicion or fever (CSF 2nd)** • **Abnormal baseline results (CSF 2nd)** • **Monitoring treatment response or further diagnostic clarification (CSF 3rd)**
• CSF analysis	Including cell count, CSF/serum glucose-ratio, lactate levels and pathogen detection	Including cell count, CSF/serum glucose-ratio, lactate levels and pathogen detection
• **Correction of leukocyte cell counts in CSF for erythrocyte contamination**	**Not conducted**	**Standard **^**1**^
• Microbiological diagnostics	Culture, antibiogram, resistance testing	Culture, antibiogram, resistance testing
• Molecular diagnostics	Eubacterial PCR only when clinical indicated^2^	Eubacterial PCR only when clinical indicated^2^
• **Interpretation of CSF parameters**	**Cell count**	**Multi-parametric approach using CSF cell count, CSF/serum glucose-ratio and lactate**
• **Diagnostic criteria for ventriculitis**	**Fever, clinical symptoms, cell count**	**Four definitions of ventriculitis diagnosis were established**^**3**^
**4. ABS management**		
• ABS Team counseling	Once a week and on demand	Once a week and on demand
• **Initiation of empirical antibiotic therapy**	**Based on clinical signs and findings from the 1 st or 2nd CSF analysis**	**Based on clinical signs and findings from the 2nd CSF analysis**
• Antibiotic selection for empirical therapy	Meropenem and vancomycin	Meropenem and vancomycin
• Application mode of meropenem and vancomycin	Meropenem as a bolus followed by prolonged infusion over four hours, combined with vancomycin as a bolus following continuous infusion	Meropenem as a bolus followed by prolonged infusion over four hours, combined with vancomycin as a bolus following continuous infusion
• **Cessation of empirical antibiotic therapy**	**No de-escalation**	**If no pathogens were detected and CSF parameters normalized or significantly improved after 48–72 h**
• **Targeted anti-infective** **treatment**	**Inconsistent adjustment of antibiotic therapy**	**Pathogen-directed antibiotic therapy adjusted according to microbiological findings, including a de-escalation strategy**
• **TDM of antibiotic therapy**	**Performed at the discretion of the treating team**	**Trough serum levels 24 h after treatment initiation, then daily until target concentrations are achieved (meropenem: 20–25 mg/L; vancomycin: 25–30 mg/L)**
• **Duration of antibiotic therapy**	**Performed at the discretion of the treating team**	**In accordance with guidelines and evaluation by the ABS team**

Table rows displayed in bold indicates differences in measures before and after SOP implementation

*Abbreviations*: *ABS *antibiotic stewardship, *CSF* cerebrospinal fluid, *EVD* external ventricular drainage, *PCR* polymerase chain reaction, *SOP* standard operation procedure,* TDM* therapeutic drug monitoring

^1^Corrected Cell count = $\text{Cell count} \left(\mathrm{CSF}\right)-[\mathrm{Erys} \left(\mathrm{CSF}\right)* \frac{\mathrm{Leucocytes} \left(\mathrm{FBC}\right)}{\mathrm{Erythrocytes} \left(\mathrm{FBC}\right)*1000}]$

^2^Eubac-PCR testing was not routinely used and reserved for cases of treatment failure under antibiotic therapy

^3^Definitions of ventriculitis diagnosis are presented in Table 2

### Group classification

To evaluate the impact of the SOP implementation all eligible patients were stratified into two groups:

- Group 1 (Pre–SOP group): Patients who received EVD treatment in the ICU before SOP implementation, between January 2019 and May 2021.
- Group 2 (Post-SOP group): patients treated with an EVD after the SOP was fully implemented, from September 2021 to December 2023.

To minimize potential overlap and ensure clear temporal separation of the cohorts, a defined “wash-out” period was established between June and August 2021, during which no patient data were included in the analysis.

### Research questions

The primary objective was to assess clinical outcomes before and after SOP implementation.

The primary endpoint was the incidence rate of study-defined EVD-associated ventriculitis.

Secondary analyses evaluated the SOP’s impact on anti-infective management, including empirical antibiotic use, meropenem and vancomycin consumption, treatment duration, ICU LOS, ICU mortality and team compliance with SOP elements.

Additionally, the distribution of CSF and clinical data from confirmed and unconfirmed ventriculitis cases was stratified.

### Outcome definitions

The primary endpoint was diagnosed EVD-associated ventriculitis according to predefined study criteria (Table 2). To minimize bias inherent to the pre–post design, ventriculitis status was adjudicated retrospectively for both cohorts using identical diagnostic criteria. Case classification was independent of the treating team’s contemporaneous documentation.

**Table 2:** Definitions of ventriculitis diagnosis

**1. Infection** Confirmed ventriculitis	• One or more positive cultures + pathological CSF findings + clinical symptoms (e.g. fever) • Pathological CSF findings + clinical symptoms (e.g. fever) but no positive cultures • Multiple positive cultures without pathological CSF findings + clinical symptoms (e.g. fever)
**2. No infection**	• No pathological CSF findings + no pathogen detected + no clinical symptoms
**3. Contamination** (No infection)	• Single positive culture without pathological CSF findings + no clinical symptoms
**4. Colonization** (No infection)	• Multiple positive cultures without pathological CSF findings + no clinical symptoms

No single parameter alone establishes the diagnosis of ventriculitis; therefore, all laboratory parameters including cell count, glucose-ratio, lactate and microbiological findings must be interpreted within the clinical context

*Abbreviations*: *CSF* cerebrospinal fluid

“Confirmed ventriculitis” comprised culture-positive cases with compatible clinical and CSF findings or culture-negative cases with compatible clinical signs and pathological CSF profiles after exclusion of alternative explanations (e.g., sterile postoperative inflammation). “Not-confirmed ventriculitis” referred to cases in which empirical therapy was initiated due to clinical concern and/or abnormal index CSF parameters but subsequently discontinued when follow-up CSF trajectories (48–72 h) normalized and no pathogen was detected (Table 2). As a secondary, more objective endpoint, we additionally report culture-positive ventriculitis.

### Incidence density per 1000 EVD days

The incidence density was calculated to account for differences in exposure time and to allow a standardized comparison of infection risk per EVD day between study periods.

$$\text{Incidense density per 1000 EVD days}=\frac{\text{Number of cases}}{ \text{EVD days} }*1000$$

### Corrected cell count

For cell count analysis, leukocyte counts in hemorrhagic CSF were corrected using the following formula: *Corrected Cell count* = $\text{Cell count} \left(\mathrm{CSF}\right)-[\mathrm{Erys} \left(\mathrm{CSF}\right)* \frac{\mathrm{Leucocytes} \left(\mathrm{FBC}\right)}{\mathrm{Erythrocytes} \left(\mathrm{FBC}\right)*1000}]$

Following SOP implementation, calculations were performed systematically (Table 1). To allow cohort comparison, cell counts in the pre-SOP group were retrospectively corrected using this formula.

### CSF sampling

CSF sampling frequency was quantified to contextualize infection and contamination risk. The median number of CSF samples per patient was three in both cohorts (Table 1). Sampling followed the SOP-defined diagnostic algorithm post-implementation and was primarily triggered by clinical suspicion or abnormal baseline findings (Supplementary Fig. S1).

### Antibiotic consumption

Annual antibiotic consumption for meropenem and vancomycin among neurosurgical patients in the ICU was calculated as recommended daily doses (RDD) per 100 patient-days following the recommendation by the Robert Koch Institute (RKI) in Germany using the formula:

$$\mathrm{RDD} /100\; \mathrm{patient}-\mathrm{days}=\frac{\text{Total antibiotic amount} (\mathrm{g})}{\text{Mean recommended daily dose} (\mathrm{g}/\mathrm{day}) * \text{Total patient}-\mathrm{days} }*100$$

### Statistical analysis

Analyses were conducted per patient during the index ICU stay.

The effect of the SOP intervention on the primary endpoint was estimated in terms of an odds ratio (OR) with 95% confidence interval (CI). The probability of infection with EVD-associated ventriculitis was calculated for both groups using the Wilson score method, with 95% CI. To further evaluate the primary endpoint, Boschloo’s exact test was applied. A logistic regression with Firth’s bias correction was performed to adjust for the potential risk factors SAH and EVD duration. Given the low event rate, the number of covariates included in the model was intentionally limited to those risk factors considered most clinically. The evaluation of the endpoint must be understood as exploratory, therefore p-values do not allow confirmatory conclusions.

Standardized mean differences (SMDs) were calculated to assess the magnitude of differences between groups. As this study was conducted as a retrospective analysis without a pre-specified statistical analysis plan, SMD thresholds were not formally established a priori. Interpretation followed the conventional reference framework proposed by Cohen: SMD < 0.2 was considered negligible, 0.2–0.5 small, 0.5–0.8 moderate, and > 0.8 large. SMDs offer context for group differences in this exploratory analysis. No additional statistical tests were conducted.

The secondary analysis, as well as demographic and clinical characteristics, was analyzed descriptively. For continuous variables, descriptive statistics included the median (50th percentile), along with the lower and upper quantiles (25th–75th percentile) referred to as Q1 and Q3. For categorical variables, statistics included absolute and relative frequencies. For the secondary endpoints, OR as well as probability rates with 95% CI was calculated as well.

## Results

### Patient cohort

**Fig. 1:**
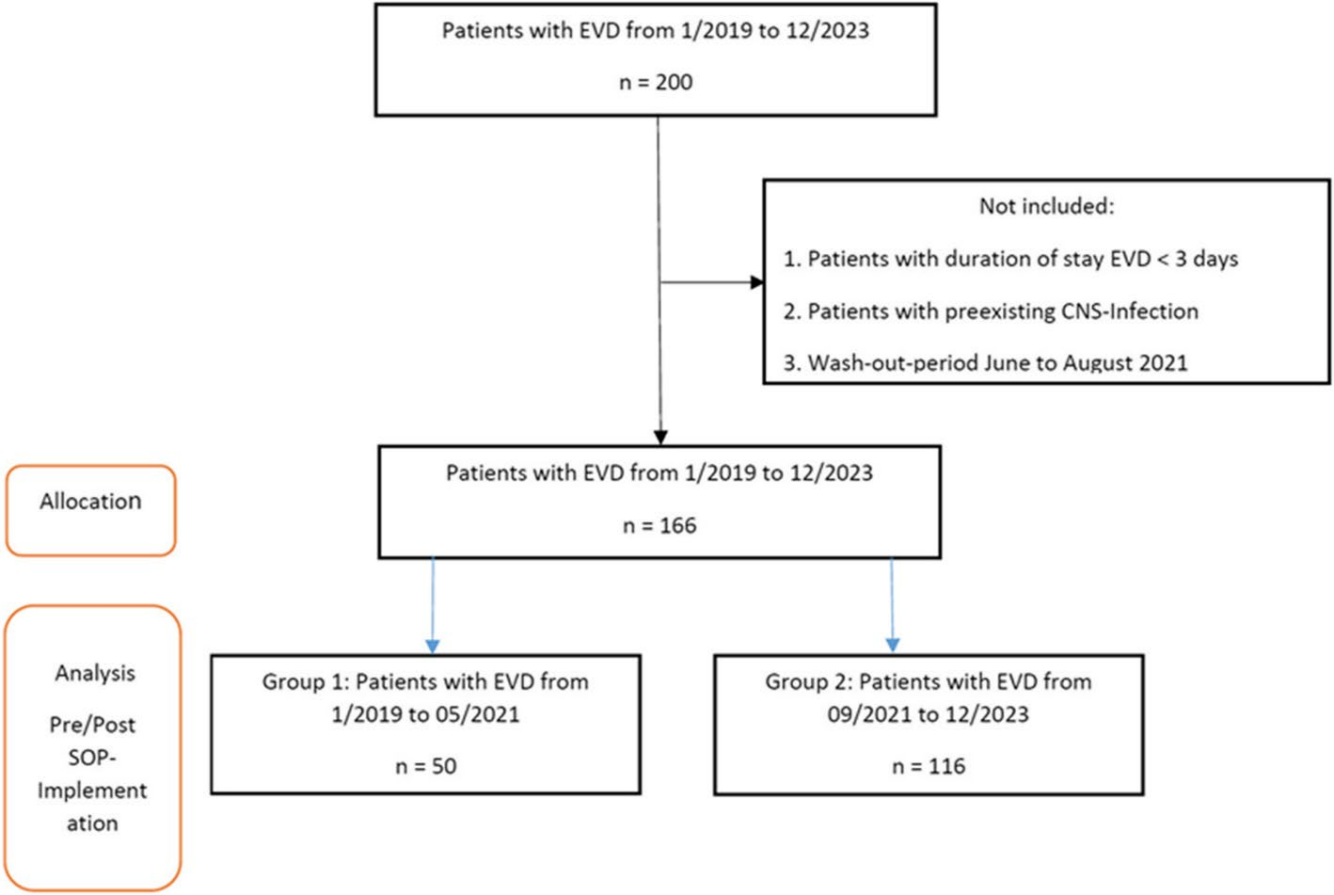
Study flow chart. A detailed visual description of the numbers of included and excluded patients. Abbreviations: EVD, External ventricular drainage, n, number, CNS, Central nervous system, SOP, Standard Operating Procedure

Between January 2019 and December 2023, a total of 200 patients received an EVD while treated in the ICU. Of these, 21 patients were excluded based on predefined criteria. Thirteen patients were treated during the designated wash-out period and were not assigned to either study group. Consequently, 166 patients were included in the final pre–post analysis (Fig. 1, Table 3).

**Table 3:** Patients characteristics

	All patients *n* = 166	Group 1 pre-SOP *n* = 50	Group 2 post-SOP *n* = 116	SMD
Age at diagnosis, median (IQR)	62 (52, 74)	62 (52, 76)	62 (52, 74)	0.130
Gender				0.109
Male, *n* (%)	80 (48.2)	26 (52.0)	54 (46.6)	
Female, *n *(%)	86 (51.8)	24 (48.0)	62 (53.4)	
Comorbidities				0.459
None, *n *(%)	**102 (61.4)**	**28 (56.0)**	**74 (63.8)**	
Autoimmune disease, *n *(%)	2 (1.2)	1 (2.0)	1 (0.9)	
COPD, *n* (%)	8 (4.8)	1 (2.0)	7 (6.0)	
Diabetes mellitus, *n *(%)	**12 (7.2)**	**7 (14.0)**	**5 (4.3)**	
Cardiovascular disease, *n *(%)	6 (3.6)	1 (2.0)	5 (4.3)	
Liver cirrhosis, *n* (%)	11 (6.6)	3 (6.0)	8 (6.9)	
Renal insufficiency, *n *(%)	3 (1.8)	1 (2.0)	2 (1.7)	
Tumour disease, *n* (%)	**22 (13.3)**	**8 (16.0)**	**14 (12.1)**	
Indication for EVD				0.397
Stroke, *n* (%)	**6 (3.6)**	**3 (6.0)**	**3 (2.6)**	
Brain tumour, *n* (%)	19 (11.4)	4 (8.0)	15 (12.9)	
Hydrocephalus, *n *(%)	2 (1.2)	0 (0)	1 (1.7)	
ICH, *n* (%)	27 (16.3)	6 (12.0)	21 (18.1)	
ICH with intraventricular extension, *n *(%)	**17 (10.2)**	**6 (12.0)**	**11 (9.5)**	
IVH, *n *(%)	3 (1.8)	1 (2.0)	2 (1.7)	
SAH, *n* (%)	**61 (36.7)**	**22 (44.0)**	**39 (33.6)**	
TBI, *n* (%)	31 (18.7)	8 (16.0)	23 (19.8)	
SAPS II-Score at admission, median (IQR)	37 (32, 46)	39 (30, 55)	37 (32, 43)	0.360
SOFA-Score at admission, median (IQR)	8 (4, 10)	8 (4, 10)	8 (4, 10)	0.089

Table rows displayed in bold indicates events with a higher incidence in Group 1 (pre-SOP)

While age, gender, and SOFA score at admission showed negligible differences in SMD calculation (SMD < 0.13), small-to-medium imbalances were observed in comorbidities (SMD = 0.459), indication for EVD (SMD = 0.397) and SAPS II score (SMD = 0.360)

*Abbreviations*: *COPD* chronic obstructive pulmonary disease, *CSF* cerebrospinal fluid, *EVD* external ventricular drainage, *ICH* Intracerebral haemorrhage,* IQR* interquartile range, *IVH* intraventricular haemorrhage, *SAB* subarachnoid haemorrhage, *SAPS* Simplified Acute Physiology Score,* SMD* Standardized Mean Difference, *SOFA* Sequential Organ Failure Assessment Score, *TBI* traumatic brain injury, *n *number

### Primary endpoint: EVD-associated ventriculitis

The overall rate of study-defined EVD-associated ventriculitis across both groups was 13.3% (Table 4). In Group 1, the rate of diagnosed infection was 22.0% (11/50) [95% CI 0.13–0.35], in Group 2, it was reduced to 9.5% (11/116) [95% CI 0.05–0.16] with an unadjusted OR 0.37 [95% CI 0.13–1.04]; *p* = 0.053.

**Table 4:** Study Results pre- and post-implementation of the SOP

	All patients *n* = 166	Group 1 pre-SOP *n* = 50	Group 2 post-SOP *n* = 116	SMD
Ventriculitis, *n *(%)	22 (13.3)	11 (22.0)	11 (9.5)	0.349
Not-confirmed ventriculitis, *n *(%)	7 (4.2)	3 (6.0)	4 (3.4)	0
Pathogen detection, *n* (%)	14 (8.4)	7 (14.0)	7 (6.0)	0.067
EVD duration, days, median (IQR)	7.0 (4.0, 11)	9.0 (5.0, 14)	6.5 (4.0, 10)	0.376
EVD duration before onset of ventriculitis, days, median (IQR)	7.0 (5.0, 10)	8.0 (4.5, 10)	6.0 (5.5, 10)	0.145
Pre-Procedural antibiotic prophylaxis, *n* (%)	144 (86.7)	38 (76.0)	106 (91.4)	0.426
Empirical antibiotic therapy, *n* (%)	29 (17.5)	14 (28.0)	15 (12.9)	0.425
Duration of antibiotic therapy among treated patients, days, median (IQR)	14 (7.0, 16.0)	16 (12.0, 18.0)	10 (6.5, 15.0)	0.796
ICU length of stay, days, median (IQR)	12 (7.0, 19.0)	15 (8.0, 22.0)	11 (7.0, 18.0)	0.268
Mortality, *n* (%)	44 (26.5)	13 (26.0)	31 (26.7)	0.016

Several outcome parameters showed clinically relevant SMDs between pre- and post-SOP groups. The duration of antibiotic therapy among treated patients demonstrated a large difference (SMD = 0.796), supporting a strong effect of the SOP on ABS. Difference in ventriculitis incidence (SMD = 0.349), represented a clinically meaningful reduction from 22.0 to 9.5%, though the SMD appears modest because it accounts for variability in the data**.** Pre-procedural antibiotic prophylaxis (SMD = 0.426) indicated a small-to-medium effect, representing improved protocol adherence (76.0% to 91.4%). Empirical antibiotic therapy (SMD = 0.425) showed a small-to-medium effect, suggesting fewer suspected infections or improved diagnostic interpretation requiring treatment (28.0% to 12.9%). ICU mortality showed no relevant difference (SMD = 0.016), indicating safety of the SOP implementation

*Abbreviations*: *CSF* cerebrospinal fluid, *EVD* external ventricular drainage, *ICU* intensive care unit,* IQR* interquartile range, *SMD* standardized mean difference, *n* number

The incidence density of study-defined ventriculitis decreased from 21.1 per 1000 EVD days in the pre-SOP period to 12.0 per 1000 EVD days in the post-SOP period, corresponding to a relative reduction of approximately 43%. Similarly, the incidence density of culture-positive ventriculitis declined from 13.4 to 7.6 per 1000 EVD days, also representing a relative reduction of approximately 43% (Table 5).

**Table 5:** Incidence density per 1000 EVD days of study-defined ventriculitis and culture positive ventriculitis

Outcome	Group 1 pre-SOP *n* = 50	Group 2 post-SOP *n* = 116
Study-defined ventriculitis		
n/EVD days	11/521	11/919
Study-defined ventriculitis Incidence density (per 1000 EVD days)	21.1	12.0
Culture positive ventriculitis		
n/EVD days	7/521	7/919
Culture positive ventriculitis Incidence density (per 1000 EVD days)	13.4	7.6

The table describes that despite identical absolute case numbers in both periods, the extended exposure time in the post-SOP period resulted in a substantially lower infection rate when adjusted for EVD duration. A relative reduction of 43% in incidence density was observed for both study-defined and culture-positive ventriculitis between the pre-SOP and post-SOP periods

*Abbreviations*: *EVD* external ventricular drainage,* SOP* standard operation procedure*, n* number

By adjusting for SAH and EVD duration, we aimed to minimize bias and improve the validity of comparisons between the two groups. After adjustment for potential risk factors, the association between group assignment and infection risk was attenuated, with an adjusted OR of 0.50 [95% CI 0.19–1.35]; *p* = 0.169 (Supplementary Table S2).

### Secondary analysis

#### Antibiotic use

In Group 1, 28% [95% CI 17.5–41.7] of patients received empirical antibiotic therapy, compared to only 12.9% [95% CI 8.0–20.2] in Group 2, corresponding to an unadjusted OR of 0.38 [95% CI 0.17–0.87] and representing a relative reduction of more than 50% (Table 4). Among treated patients the median duration of antibiotic therapy decreased from 16 days [95% CI 13.5–18.5] in Group 1 to 10 days [95% CI 6.5–13.5] in Group 2, corresponding to a reduction of 6 days in the post-intervention cohort (Table 4).

The consumption of meropenem and vancomycin among neurosurgical patients in the ICU, calculated for the years 2019 to 2023 is presented in Table 6. Meropenem consumption decreased continuously over the observation period and stabilized in 2022 and 2023, reaching approximately 75% of the baseline consumption in 2019. Vancomycin consumption showed annual fluctuations over the study period.

**Table 6:** Consumption of meropenem and vancomycin

	2019	2020	2021	2022	2023
Meropenem, RDD/100 PD	30.2	28.9	26.2	19.9	20.4
Vancomycin, RDD/100 PD	16.4	19.4	15.4	18.9	15.1

The table presents the consumption per year from 2019 to 2023 calculated for all neurosurgical patients at ICU, expressed as recommended daily doses per 100 patient-days (RDD/100 PD). The average daily dose of meropenem was 6 g/day; of vancomycin 3.3 g/day

*Abbreviations*: *PD* patient days, *RDD* recommended daily dose, *n *number

The spectrum of confirmed pathogens isolated in cases of ventriculitis is summarized in the Supplementary Table S1.

### Clinical outcome

The median ICU LOS was reduced by 4 days after SOP implementation from 15.0 (IQR 8.0–22.0) in Group 1 to 11.0 (IQR 7.0–18.0) days in Group 2 (Table 4). Patients diagnosed with ventriculitis experienced a longer ICU LOS as compared to patients with no ventriculitis in both groups:

- Group 1: 22.0 vs. 6.0 days (IQR 22.0–25.0 vs. IQR 5.0–10.0)
- Group 2: 19.0 vs. 6.0 days (IQR 15.0–25.0 vs. IQR 4.0–8.0)

These findings show a reduction in ICU LOS of up to 16 days in Group 1 and 13 days in Group 2 in patients without ventriculitis.

### ICU mortality

There was no difference in ICU mortality (Table 4): Group 1: 26% (13/50), [95% CI 0.16–0.4] and Group 2: 27% (31/116), [95% CI 0.20–0.35]; the corresponding OR was 0.91 [95% CI 0.46–2.42].

### SOP adherence of the treatment team

Administration of antibiotic prophylaxis prior to EVD insertion improved from 76% pre-SOP to 91.4% post-SOP. The rate of empirical antibiotic therapy initiation based on the algorithm decreased from 28% in Group 1, to 12.9% in Group 2.

Adjustment of antibiotic therapy including early discontinuation in non-confirmed ventriculitis and pathogen-directed modification was observed in 35.7% (5/14) of empirically treated cases in Group 1 and 40% (6/15) in Group 2.

The detailed results are summarized in Table 7.

**Table 7:** SOP adherence of the treatment team

	All patients *n* = 166	Group 1 pre-SOP *n* = 50	Group 2 post-SOP *n* = 116
1 st CSF examinations, *n* (%)	100 (60.2)	27 (54.0)	73 (62.9)
2nd CSF examinations, *n *(%)	112 (67,5)	31 (62.0)	81 (69.8)
3rd CSF examinations, *n *(%)	43 (25.9)	16 (32.0)	27 (23.3)
Preoperative antibiotic prophylaxis,			
*n* (%)	144 (86.7)	38 (76.0)	106 (91.4)
Empirical antibiotic therapy, *n *(%)	29 (17.5)	14 (28.0)	15 (12.9)
Empirical antibiotic therapy	All patients	Group 1 pre-SOP	Group 2 pre-SOP
	*n* = 29	*n* = 14	*n* = 15
Adjustment after pathogen detection, *n* (%)	11 (37.9)	5 (35.7)	4 (26.7)
Termination in not-confirmed ventriculitis, *n* (%)	2 (6.7)	0	2 (13.3)
No adjustment, *n *(%)	3 (10.0)	2 (14.3)	1 (6.7)

The adherence of the treatment team to the SOP was analyzed regarding the following key aspects: frequency of csf sampling, administration of antibiotic prophylaxis prior EVD insertion, initiation of empirical antibiotic therapy based on the algorithm, adjustment of antibiotic therapy according to follow-up investigations and premature discontinuation of antibiotic therapy in not-confirmed ventriculitis or adjustment of antibiotic therapy after pathogen identification

*Abbreviations*: *CSF* cerebrospinal fluid, *SOP* standard operation procedure, *n* number

### Additional analyses

#### Impact of intraventricular blood accumulation on infection rate

In a cross-group comparison, patients with intraventricular blood accumulation experienced an infection rate of 18.5% (15/81) [95% CI: 0.11–0.27]. In contrast, patients without intraventricular blood accumulation, e.g., stroke, brain tumours, showed a lower infection rate of 8.2% (9/95) [95% CI 0.04–0.16]. The calculated OR was 2.07 [95% CI 0.9–7.76], indicating that the risk of infection rate in patients with intraventricular blood accumulation was twice as high; however, the wide confidence interval suggests that this association is not statistically significant (Supplementary Table S2).

## Discussion

This retrospective pre–post study suggests that implementation of a multifaceted interdisciplinary SOP was associated with a numerical reduction in study-defined (22.0% to 9.5%) and culture-positive (14.0% to 6.0%) EVD-associated ventriculitis, halved rates of empirical antibiotic initiation (28.0% to 12.9%) and shorter treatment duration (16 days to 10 days). However, it must be emphasized that the primary endpoint did not reach statistical significance: the unadjusted OR was 0.37 [95% CI 0.13–1.04]; *p* = 0.053, with the confidence interval crossing 1.0. After adjustment for EVD duration and SAH, the association was further attenuated (adjusted OR 0.50 [95% CI 0.19–1.35]; *p* = 0.169). These results are consistent with a clinically meaningful trend but do not constitute statistical proof of a treatment effect. The limited number of events (22 cases in 166 patients) substantially restricted statistical power, and the findings should be interpreted as hypothesis-generating rather than confirmatory. Despite reduced antimicrobial exposure, ICU mortality remained unchanged, while ICU LOS decreased by 4 days, particularly among patients without ventriculitis. These findings suggest improved infection control and antimicrobial stewardship without evidence of adverse clinical outcomes, though causal inference is not possible given the study design.

To account for differences in catheter exposure time, incidence density was calculated as events per EVD day, enabling standardized comparison between study periods. Both study-defined and culture-positive ventriculitis showed an approximately 43% relative reduction in the post-SOP period, suggesting improved infection control relative to catheter duration.

Analyses were further adjusted for established risk factors, including EVD duration and subarachnoid SAH, which has been linked to increased susceptibility to infection through intraventricular blood-associated inflammatory responses [4, 12, 13]. Median EVD duration was longer in the pre-SOP cohort (9 days pre-, 6.5 days post-SOP), potentially reflecting delayed drain removal. Logistic regression with Firth correction yielded an adjusted odds ratio of 0.5 for ventriculitis in the post-SOP group, consistent with a substantial risk reduction, although confidence intervals remained wide due to the limited number of events.

Device-associated ventriculitis remains a diagnostic and therapeutic challenge due to the absence of standardized criteria and universally accepted gold standard. Definitions vary in CSF parameters, threshold values and adjunctive diagnostics [1, 14, 15]. Karvouniaris et al. compared IDSA and CDC criteria with various studies, illustrating the lack of reproducible diagnostic standards [16]. Current diagnosis typically relies on abnormal CSF findings, pathogen detection, neurological deterioration and systemic or local signs of infection [1, 14]. Among these, CSF-chemistry is particularly important in early diagnosis [17, 18]. Clinical symptoms such as fever, meningism and altered mental status are nonspecific and may result from the underlying neurologic insult [5]. Culture-based pathogen detection is limited by delayed turnaround (2–4 days) and poor sensitivity (20–50%), even in patients with abnormal CSF results [19, 20]. Moreover, aseptic inflammation caused by haemorrhage or foreign material can mimic infection by elevating CSF cell counts without microbial aetiology, leading to potential overdiagnosis [4, 12, 14]. This diagnostic ambiguity frequently prompts the initiation of empirical antibiotic therapy, driven by the high morbidity and mortality associated with ventriculitis-related complications [7]. Reported in-hospital mortality rates range from 15 to 23% in affected patients [7] and may reach 30% in patients with severe complications such as brain abscess or empyema [19].

To address these challenges, we developed an interdisciplinary SOP tailored to local practices and based on international guidelines. Previous studies showed that IPC bundles, including aseptic insertion and catheter care can reduce ventriculitis rates [20–24]. Zheng et al. proposed a diagnostic algorithm based on standard CSF parameters with 95.3% specificity and 88.6% accuracy [25]. However, evidence on the impact of such algorithms on antimicrobial use has been lacking. Our SOP was designed to enhance standardization of the diagnostic workup and interpretation of CSF findings across teams. Before SOP implementation, diagnosis was mainly based on CSF cell counts and clinical signs. As the reliance on a single-parameter yields sensitivities of only 60% [4, 14, 17], the SOP introduced a multiparametric diagnostic approach integrating CSF cell count (including correction for erythrocyte contamination), CSF/serum glucose ratio, and lactate, supported by serial CSF analyses and clinical assessment [10, 11, 15–17, 26]. Correction of leukocyte cell counts for erythrocyte contamination may further improve diagnostic accuracy [10, 11, 15]. Since defined cut-off values for nosocomial CNS infections are lacking, serial CSF analyses and their temporal trajectories together with clinical judgement remain essential [17]. CSF parameters and clinical signs were summarized descriptively across clinically classified categories (no ventriculitis, confirmed ventriculitis, and not-confirmed ventriculitis), (Table 8). These distributions are reported to characterize observed patterns within the cohort and should not be interpreted as defining diagnostic thresholds or implying diagnostic performance. In the absence of an independent reference standard and given the non-randomized design, the present study does not support formal statements on diagnostic test performance or causal effects. Rather, the observed changes likely reflect reduced diagnostic variability and more uniform interpretation of CSF findings across clinical teams.

**Table 8:** Descriptive summary of the recorded clincal signs and CSF values

	All patients *n* = 166	No ventriculitis *n* = 137	Ventriculitis confirmed *n* = 22	Ventriculitis not-confirmed *n* = 7
Fever^1^, *n *(%)	96 (57.8)	74 (54.0)	19 (86.4)	3 (42.9)
Leukocytes^2^,/nL,	11,000	11,000	13,000	12,000
median (IQR)	(8800, 15,000)	(8400, 14,000)	(11,000, 18,000)	(11,000, 19,000)
1 st CSF examination^3^, median (IQR)				
Cell count,/µL	65 (19, 210)	72 (22, 250)	60 (11, 140)	12 (3.5, 28)
CSF/serum Glucose-ratio	0.60 (0.50, 0.70)	0.60 (0.40, 0.70)	0.60 (0.50, 0.70)	0.65 (0.58, 0.70)
Lactate, mmol/L	3.3 (2.6, 4.4)	3.3 (2.6, 4.6)	3.3 (3.0, 4.3)	3.1 (2.6, 3.4)
2nd CSF examination^4^, median (IQR)				
Cell count,/µL	**65 (17, 290)**	**56 (12, 170)**	**440 (110, 1500)**	**84 (40, 100)**
Corrected cell count,/µL	**54 (14, 180)**	**46 (12, 100)**	**380 (89, 1300)**	**24 (5, 72)**
CSF/serum Glucose-ratio	**0.50 (0.4, 0.6)**	**0.60 (0.5, 0.7)**	**0.40 (0.33, 0.50)**	**0.50 (0.4, 0.60)**
Lactate, mmol/L	**3.2 (2.5, 4.4)**	**2.9 (2.4, 3.7)**	**4.8 (4.4, 6.2)**	**3.3 (3.1, 4.3)**
Pathological				
2nd CSF examination, *n *(%)	17 (10.2)	0 (0)	17 (77.3)	0 (0)
Pathogen detection, *n* (%)	14 (8.4)	0 (0)	12 (54.5)	2 (28.6)
3rd CSF examination^5^, median (IQR)				
Cell count,/µL	74 (28, 120)	74 (32, 120)	74 (20, 110)	40 (37, 120)
Corrected cell Count,/µL	49 (16, 89)	53 (11, 77)	42 (17, 90)	40 (37, 54)
CSF/serum Glucose-ratio	0.50 (0.50, 0.60)	0.60 (0.50, 0.63)	0.50 (0.50, 0.60)	0.50 (0.40, 0.50)
Lactate, mmol/L	3.3 (2.8, 3.9)	3.2 (2.7, 3.8)	3.6 (3.0, 4.9)	3.3 (3.1, 3.8)

Cerebrospinal fluid parameters are descriptively presented across clinically defined categories, including no ventriculitis, confirmed ventriculitis, and retrospectively unconfirmed ventriculitis after follow-up diagnostics. The 2nd CSF analysis is the index examination used to assess an infection; the results are shown in bold

*Abbreviations*: *n* number, *CSF *cerebrospinal fluid

^1^Fever was defined as an increase in body temperature above 37.8 °C

^2^Leukocytes in peripheral blood were considered elevated at > 9,000/µl

^3^ 1 st CSF examination: Baseline at EVD insertion

^4^2nd CSF examination: Index CSF Analysis: 2nd CSF examination is performed if there is suspicion of infection or if 1 st CSF examination shows abnormal findings. This serves as a reference point to assess changes in CSF parameters and helps determine the need for further diagnostic or therapeutic steps

^5^ 3rd CSF examination: treatment response or further diagnostic clarification

A key finding was the reduction in empirical antibiotic initiation and shorter treatment duration in the post-SOP cohort. Structured interdisciplinary review of CSF parameters, microbiological findings, and antimicrobial regimens likely facilitated more consistent de-escalation and pathogen-directed therapy [1, 18, 27, 28]. Empirical antibiotic treatment was initiated by the second CSF analysis and clinical context (fever, neurological deterioration). Although frequent CSF sampling has been associated with an increased risk of infection [29], the second CSF examination was performed post-SOP more consistently, as it was considered by the clinical team—together with pathogen detection—to be a key indicator for the initiation of empirical antibiotic therapy.

In confirmed ventriculitis, antibiotic treatment was associated with declining CSF leukocyte counts and lactate levels, increasing CSF/serum glucose ratios and clinical improvement within 48–72 h. In culture-positive cases (54%), therapy was adjusted according to susceptibility testing, whereas in culture-negative cases treatment was continued only when abnormal CSF findings and clinical suspicion persisted. Transient aseptic inflammatory changes frequently resolved without sustained antimicrobial therapy, underscoring both the value of serial CSF monitoring and the limitations of conventional culture-based diagnostics [4, 14, 19, 20].

Although Polymerase chain reaction (PCR)-based methods may improve pathogen detection in selected cases, their performance remains limited for common Gram-positive, skin flora-associated pathogens such as *Staphylococcus epidermidis* and *Cutibacterium acnes* [30, 31]. These organisms accounted for the majority of culture-confirmed ventriculitis cases in the present cohort. In our diagnostic algorithm, Eubac-PCR testing was reserved for cases of treatment failure under antibiotic therapy.

The annual antibiotic consumption was presented for the neurosurgical patients on the ICU, assessed as RDD per 100 patient-days in accordance with the RKI national guidelines. The calculation of RDD is based on the mean *recommended* daily dose (rather than the *defined* daily dose, which reflects an average daily dose) which more accurately represents dosing practices in German hospitals and enables standardized comparisons at the national level. Meropenem consumption showed a downward trend prior to full SOP implementation, with a more pronounced decline observed after SOP introduction, associated with a more consistent discontinuation of empirical therapy in not-confirmed ventriculitis. Vancomycin consumption remained unchanged, concordant with the predominance of Gram-positive pathogens (81.3%, mainly *Staphylococcus epidermidis* and *Cutibacterium acnes)* which are skin flora-associated organisms commonly associated with EVD-related infection*s* [32].

Prior to SOP implementation, EVD-related practices were not formalized within a single interdisciplinary protocol. The SOP was introduced through a structured implementation phase including structural staff training and ongoing clinical support. After implementation, CSF sampling, EVD management and antimicrobial regimens were reviewed more consistently during interdisciplinary and antimicrobial stewardship rounds. The standardization improved clarity and adherence, reflected by higher rates of preoperative prophylaxis (76.0% to 91.4%), more uniform CSF assessment, reduced initiation of empirical antibiotics and more frequent therapy adjustments guided by clinical and microbiological findings.

While overall adherence improved in several key areas, adherence to certain SOP components remained suboptimal in the post-SOP period. Antibiotic therapy adjustment after pathogen detection was documented in only 26.7% of empirically treated patients in Group 2, and early termination of therapy in not-confirmed ventriculitis occurred in only 13.3% of cases. These findings indicate that de-escalation and pathogen-directed therapy adaptation, although structurally embedded in the SOP, were inconsistently implemented in clinical practice. Possible reasons include physician uncertainty regarding early discontinuation in the context of ongoing clinical instability and the complexity of antimicrobial decision-making in critically ill neurosurgical patients, e.g. planning ventricular shunt implantation. These gaps represent important targets for future quality improvement efforts and underscore the need for sustained education, structured feedback, and prospective adherence monitoring. They may also have attenuated the measurable effect of the SOP on antimicrobial stewardship outcomes.

### Limitations

Firstly, it was conducted at a single-centre, which may limit generalizability. Secondly, its retrospective design depended on documentation quality and excluded outcomes beyond ICU discharge. Thirdly, unequal group sizes, influenced by the COVID-19 pandemic, may have introduced bias. Fourthly, the low incidence of study-defined ventriculitis limited statistical power, allowing only a trend toward benefit. Fifth, lumbar CSF drainage was excluded. Sixthly, TDM was implemented and interpreted consistently only after SOP introduction; pre-SOP TDM data were incomplete and therefore not analyzed. Seventhly, detailed analysis of neurosurgical interventions such as EVD exchange frequency or endoscopic intraventricular lavage was not feasible due to incomplete documentation and limited event numbers.

## Conclusions

This study describes the development and implementation of an interdisciplinary SOP for the prevention, diagnosis, and management of nosocomial EVD-associated ventriculitis and its association with observed infection rates and patterns of antimicrobial use. In the absence of an independent reference standard or blinded adjudication, no inferences regarding diagnostic test performance or causal effects can be made. Instead, the findings indicate reduced diagnostic variability, a more uniform interpretation of cerebrospinal fluid parameters, and comparatively lower and more consistent patterns of anti-infective utilization across clinical teams.

Future multicentre prospective studies with independent adjudication and predefined endpoints are warranted to validate SOP components and quantify effects on patient-centred outcomes and antimicrobial use.

## Supplementary Information

Supplementary Material 1: Figure S1. Diagnostic-therapeutic Algorithm of the SOP for the Prevention, Diagnosis and Treatment of Device-associated nosocomial Ventriculitis. Supplementary Table S1. Confirmed Pathogen Spectrum in Ventriculitis. Supplementary Table S2. Results of the Firth-corrected logistic regression including the covariates EVD duration and subarachnoid haemorrhage (SAH).

## Data Availability

The datasets generated during and/or analysed during the current study are available from the corresponding author upon reasonable request.

## Abbreviations

ABS: Antibiotic stewardship
CI: Confidence interval
COPD: Chronic obstructive pulmonary disease
CNS: Central nervous system
CSF: Cerebrospinal fluid
EVD: External ventricular drainage
ICH: Intracranial haemorrhage
ICU: Intensive care unit
IPC: Infection and prevention control
IQR: Interquartile range
IVH: Intraventricular haemorrhage
LOS: Length of stay
OR: Odds ratio
PCR: Polymerase chain reaction
RDD: Recommended daily dose
RKI: Robert Koch Institute
SAH: Subarachnoid haemorrhage
SAPS: Simplified Acute Physiology Score
SMD: Standardized mean difference
SOFA: Sequential Organ Failure Assessment Score
SOP: Standard operating procedure
SQL: Structured query language
TBI: Traumatic brain injury
TDM: Therapeutic drug monitoring
