# Supplementary material for: Impact of an interdisciplinary standard operating procedure on external ventricular drainage-associated ventriculitis and antibiotic use in intensive care unit patients: a retrospective pre–post study
Source: J Anesth Analg Crit Care. 2026 May 23;6:77. doi: 10.1186/s44158-026-00409-4 (PMC13202821; doi:10.1186/s44158-026-00409-4)
Supplement: Supplementary file 1 — Supplementary Material 1: Figure S1. Diagnostic-therapeutic Algorithm of the SOP for the Prevention, Diagnosis and Treatment of Device-associated nosocomial Ventriculitis. Supplementary Table S1. Confirmed Pathogen Spectrum in Ventriculitis. Supplementary Table S2. Results of the Firth-corrected logistic regression including the covariates EVD duration and subarachnoid haemorrhage (SAH). [file 44158_2026_409_MOESM1_ESM.docx]

**Additional file Figure 1: Diagnostic-therapeutic Algorithm of the SOP for the Prevention, Diagnosis and Treatment of Device-associated nosocomial Ventriculitis**

**
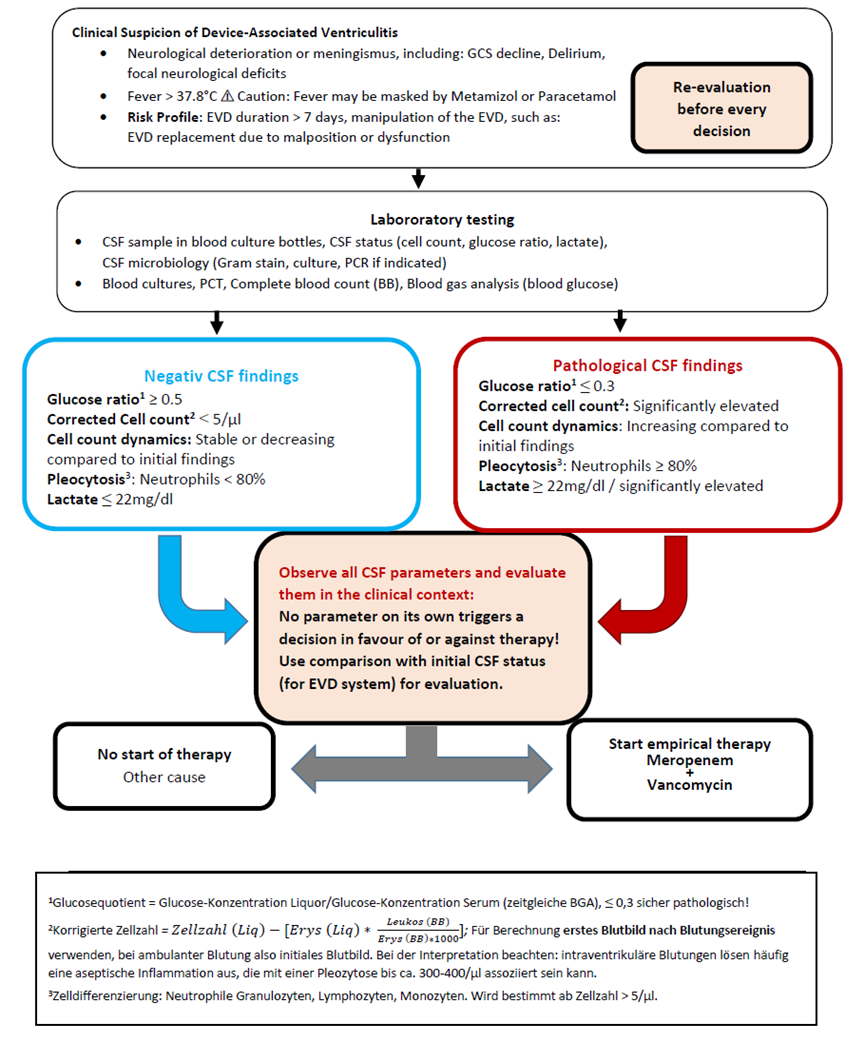
**

Lactate mg/dl in mmol/l: 22mg/dl = 2.4 mmol/l

**Additional file Table 1: Confirmed Pathogen Spectrum in Ventriculitis**

|  | Pathogens | Incidence, n |
| --- | --- | --- |
| Grampositive germs  81.3% | Staphylococcus epidermidis | 5 |
|  | Staphylococcus capitis | 1 |
|  | Staphylococcus aureus | 1 |
|  | Streptococcus constellatus | 1 |
|  | Cutibacterium acnes | 3 |
|  | Bacillus cereus | 2 |
| Gramnegative germs  18.8% | Escherichia coli | 1 |
|  | Enterobacter cloacae | 1 |
|  | Klebsiella pneumoniae | 1 |

The table describes the distribution of detected pathogens regardless of group affiliation, highlighting that gram-positive bacteria were the most common, making up 81.3% of the findings. *Staphylococcus epidermidis* being the most frequently detected pathogen (Group 1: 42.9%, Group 2: 28.6%).

Gram-negative bacteria were detected in 18.8% of cases, predominantly in patients with traumatic brain injury.

**Abbreviations:** n – number

**Additional fileTable 2: Results of the Firth-corrected logistic regression including the covariates EVD duration and subarachnoid haemorrhage (SAH)**

|  | OR | Lower 95% CI | Upper 95% CI | P-value |
| --- | --- | --- | --- | --- |
| Intercept | 0.05 | 0.02 | 0.16 | <0.001 |
| Group 2 | 0.5 | 0.19 | 1.35 | 0.169 |
| SAH | 2.31 | 0.87 | 6.32 | 0.093 |
| EVD duration | 1.11 | 1.05 | 1.19 | 0.001 |

Adjustment for the covariables subarachnoid haemorrhage and EVD duration

**Abbreviations:** EVD, External ventricular drainage; SAH, Subarachnoid haemorrhage
